# Amifostine and Melatonin Prevent Acute Salivary Gland Dysfunction 10 Days After Radiation Through Anti-Ferroptosis and Anti-Ferritinophagy Effects

**DOI:** 10.3390/ijms252111613

**Published:** 2024-10-29

**Authors:** Ji-Min Kim, Dong-Hyun Kim, Won-Taek Kim, Sung-Chan Shin, Yong-il Cheon, Gi-Cheol Park, Hyoun-Wook Lee, Byung-Joo Lee

**Affiliations:** 1 Pusan National University Medical Research Institute, Pusan National University School of Medicine, Pusan National University, Yangsan 50612, Republic of Korea; 2 Department of Radiation Oncology, Pusan National University School of Medicine, Pusan National University, Yangsan 50612, Republic of Korea; 3 Biomedical Research Institute, Pusan National University Hospital, Busan 49241, Republic of Korea; 4 Department of Otorhinolaryngology-Head and Neck Surgery, Pusan National University School of Medicine, Pusan National University, Yangsan 50612, Republic of Korea; 5 Department of Otolaryngology-Head and Neck Surgery, Samsung Changwon Hospital, Sungkyunkwan University School of Medicine, Changwon 51353, Republic of Korea; 6 Department of Pathology, Samsung Changwon Hospital, Sungkyunkwan University School of Medicine, Changwon 51353, Republic of Korea

**Keywords:** salivary gland dysfunction, xerostomia, ferritinophagy, ferroptosis, amifostine, melatonin

## Abstract

Irradiation of the head and neck inevitably leads to decreased salivary gland function. It is postulated that radiation generates excessive reactive oxygen species (ROS) and reduces salivary gland function by ferroptosis, a new cell death mechanism; however, research in this area is currently lacking. In this study, we investigated the effects of amifostine and melatonin on acute salivary gland dysfunction and ferroptosis. Thirty-two Sprague Dawley rats were divided into four groups: control, radiation, radiation + amifostine, and radiation + melatonin. ROS; iron levels; glutathione peroxidase 4; 4-hydroxynonenal; various cytokines; and fibrosis and salivary gland functional markers were measured. Western blotting was used to detect ferritinophagy. After irradiation, we observed an increase in iron levels, ROS generation, oxidized glutathione, lipid peroxidation, fibrosis, and salivary gland dysfunction and a decrease in glutathione peroxidase 4 in salivary gland tissue. Treatment with amifostine or melatonin decreased the ferroptotic response and improved acute salivary gland function 10 days after radiation. The increase in iron levels associated with ferritinophagy was reduced after treatment with amifostine or melatonin. Our results demonstrate that radiation-induced acute salivary gland dysfunction is associated with ferroptosis and ferritinophagy. Amifostine and melatonin inhibit radiation-induced ferroptosis and ferritinophagy in the salivary gland and prevent acute salivary gland dysfunction 10 days after radiation.

## 1. Introduction

Radiotherapy is an important treatment modality for several head and neck cancers [1], however, this inevitably causes salivary gland dysfunction which reduces salivary secretion from the parotid and submandibular glands and results in xerostomia (dry mouth). Radiation-induced xerostomia causes oral infection, tooth loss, impaired swallowing and speech, and taste disorders which greatly reduce the patient’s quality of life even after full recovery from head and neck cancer [2].

Radiation causes direct DNA and protein damage and also has an indirect effect through excessive generation of reactive oxygen species (ROS) [3]. Excessive ROS attenuates the antioxidant capacity of cells through oxidative stress, which further leads to severe cellular damage [4]. Salivary gland cell damage and death owing to excessive ROS generation are thought to be important mechanisms underlying radiation-induced salivary gland dysfunction. Therefore, the development of novel radioprotectants that reduce the side effects of radiation has mainly focused on the suppression of oxidative stress.

Amifostine scavenges the excess ROS generated by radiation [5,6] and is a Food and Drug Administration approved drug that prevents irradiation-induced salivary gland dysfunction [6,7]. Amifostine has limited clinical use owing to its high cost and adverse reactions, including nausea and vomiting, low blood pressure, lethargy, chills, and allergic symptoms [8]. However, there have been no reports of direct side effects on salivary gland cells. Recent studies have investigated how to prevent radiation-induced salivary gland dysfunction using compounds that have scavenging activity to remove ROS and low toxicity [9,10,11].

Ferroptosis is a recently identified novel cell death mechanism characterized by the iron-dependent accumulation of lipid ROS [12] and has been proposed as a mechanism for tissue damage caused by irradiation [3]. Ferroptotic death is morphologically, biochemically, and genetically distinct from apoptotic and non-apoptotic death owing to the central involvement of iron-dependent lipid ROS accumulation [12]. An increase in intracellular iron generates a large number of free radicals via the Fenton reaction, therefore, the level of iron is an important factor in ferroptosis [12]. However, no study has clearly identified ferroptosis and iron homeostasis as mechanisms of radiation-induced salivary gland dysfunction. This study aimed to investigate whether ferroptosis is the mechanism underlying radiation-induced acute salivary gland dysfunction. The effects of amifostine and melatonin, which are known to have ROS scavenging activity, on the inhibition of ferroptosis and recovery of salivary gland function 10 days after irradiation were evaluated.

## 2. Results

### 2.1. Gross and Histology of Tissue After Radiation

**Figure 1:**
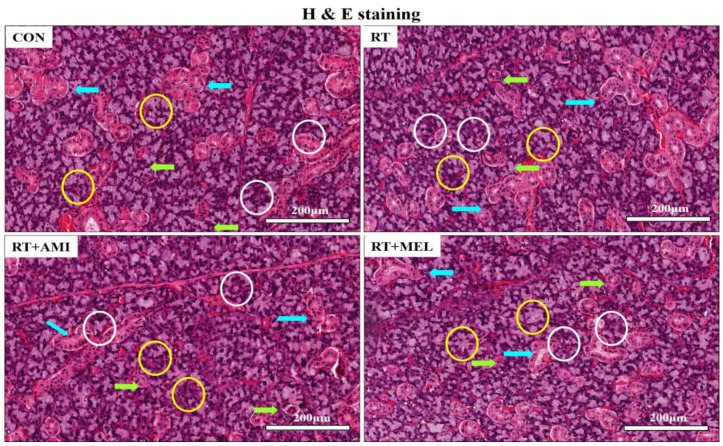
Histology of submandibular gland. The submandibular glands were mainly made up of mixed seromucous secretory units that consist of mucous acini (yellow circle) with serous demilunes (white circle). The intercalated (green arrows) and striated ducts (blue arrows) were also easily recognizable in the submandibular glands. No inflammatory cell infiltration, cellular damage, or reactive changes were observed in all groups.

Hematoxylin and eosin staining was performed to detect general morphological alterations. The submandibular glands were mainly made up of mixed seromucous secretory units that consist of mucous acini capped by semilunar-shaped serous acini. The intercalated and striated ducts were also easily recognizable in the submandibular glands. In the control and all experimental groups, the acinar and ductal systems of the submandibular glands did not show inflammatory cell infiltration or reactive morphological changes at all. Our results showed that histologically, radiation did not cause any inflammation, cellular damage, or reactive changes in the submandibular glands of the rats (Figure 1).

### 2.2. Anti-Ferroptotic Effect of Amifostine and Melatonin After Radiation Exposure

**Figure 2:**
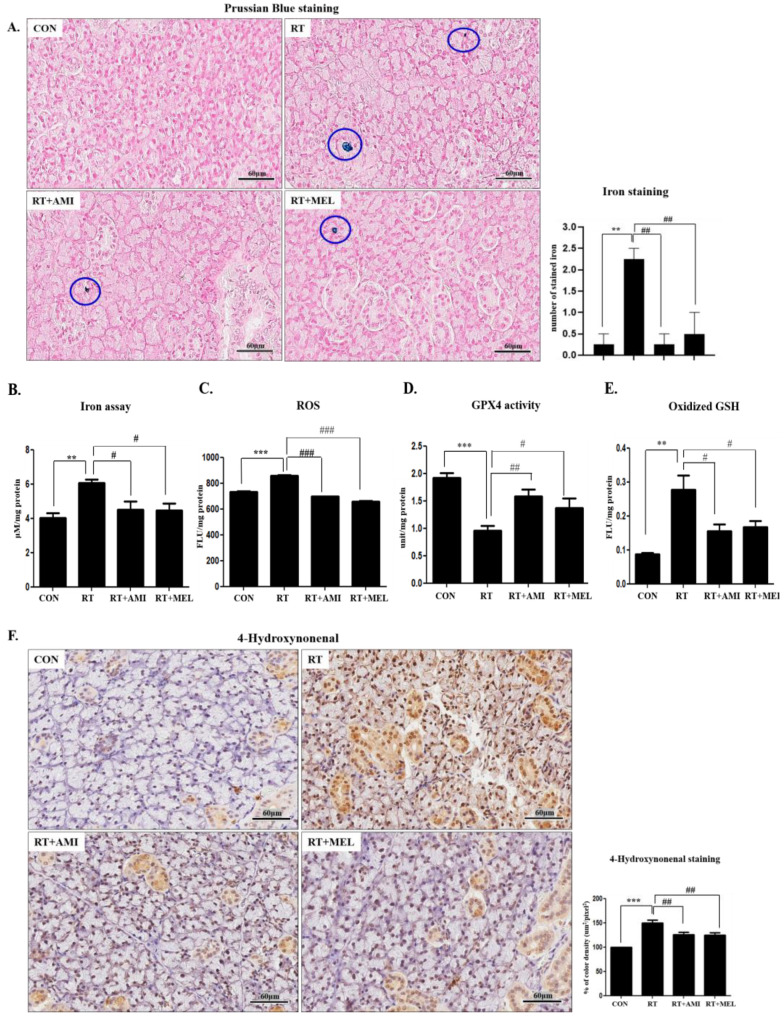
Inhibitory effect of AMI and MEL on ferroptosis in the submandibular gland was increased in the RT group but reduced following AMI and MEL treatment. (**A**) The number of stained irons of submandibular gland tissue in the RT group was higher compared with the CON group (*p* < 0.01). The number of stained irons was lower in the RT+AMI and RT+MEL groups compared with the RT group (*p* < 0.01). (**B**) Cellular total iron levels showed a significant increase in the RT group compared with the CON group (*p* < 0.01) and showed a significant decrease in the RT+AMI and RT+MEL groups compared with the RT group (*p* < 0.05). (**C**) Cytosolic ROS generation was significantly higher in the RT group compared with the CON group (*p* < 0.001) but was significantly lower in the RT+AMI and RT+MEL groups compared with the RT group (*p* < 0.05). (**D**) GPX4 activity was significantly reduced in the RT group (*p* < 0.001 compared with RT), whereas it was significantly increased in the RT+AMI (*p* < 0.01) and RT+MEL (*p* < 0.05) groups compared with the RT group. (**E**) Oxidized GSH (GSSG) concentrations in the submandibular gland increased in the RT group (*p* < 0.001), whereas they decreased in the RT+AMI and RT+MEL groups compared with the RT group (*p* < 0.05). (**F**) 4-hydroxynonenal-positive stained regions were significantly higher in the RT group (*p* < 0.001 compared to the RT group) but significantly lower in the RT+AMI and RT+MEL groups compared with the RT group (*p* < 0.001 one-way ANOVA); ** *p* < 0.01, *** *p* < 0.001 vs. CON, # *p* < 0.05, ## *p* < 0.01, and ### *p* < 0.001 vs. RT.

The observed increases in iron staining and cellular levels, ROS generation, oxidized GSH, and lipid peroxidation, coupled with the decrease in GPX4 in submandibular gland tissue after irradiation, suggest that radiation-induced submandibular dysfunction is attributable to ferroptosis. To explore whether radiation is associated with iron overload, we measured stained iron in tissue (Figure 2A). Iron was deposited in the intercellular space. The number of iron stained with blue circles in the submandibular gland tissue of the RT group was significantly higher than that of the CON group (0.25 ± 0.5, *p* < 0.01). However, the number of stained irons was significantly lower in the RT+AMI (0.25 ± 0.5) and RT+MEL (0.5 ± 1.0) groups compared with the RT group (*p* < 0.01). Notably, treatment with amifostine or melatonin resulted in decreased cellular iron levels, ROS production, GPX4 activity, oxidized GSH, and lipid peroxidation, indicating their anti-ferroptotic activity (Figure 2B–E). The inhibitory effect of AMI and MEL on ferroptosis in the submandibular gland was evident, as cytosolic iron levels showed a significant increase in the RT group (6.05 ± 0.23 µM/mg) compared with the CON group (4.03 ± 0.29 µM/mg, *p* < 0.01) but demonstrated a significant decrease in the RT+AMI (4.52 ± 0.45 µM/mg protein) and RT+MEL (4.48 ± 0.40 µM/mg) groups compared with the RT group (*p* < 0.05). Similarly, cytosolic ROS generation was significantly higher in the RT (857.06 ± 7.03 Flu/mg) group compared with the CON group (731.35 ± 5.30 Flu/mg, *p* < 0.001), yet significantly lower in the RT+AMI (696.24 ± 3.22 Flu/mg) and RT+MEL (657.00 ± 5.33 Flu/mg) groups compared with the RT group (*p* < 0.05). Moreover, GPX4 activity was significantly reduced in the RT group (0.97 ± 0.12 unit/mg, *p* < 0.001 compared with CON), whereas it was significantly increased in the RT+AMI (1.72 ± 0.13 unit/mg, *p* < 0.01) and RT+MEL (1.42 ± 0.20 unit/mg, *p* < 0.05) groups compared with the RT group. Additionally, oxidized GSH (GSSG) concentrations in the submandibular gland increased in the RT group (0.28 ± 0.04 Flu/mg, *p* < 0.001), while they decreased in the RT+AMI (0.15 ± 0.02 Flu/mg) and RT+MEL (0.17 ± 0.02 Flu/mg) groups compared with the RT group (*p* < 0.05). Furthermore, 4-hydroxynonenal-positive stained regions were significantly higher in the RT group (*p* < 0.001 compared to the RT group) but significantly lower in the RT+AMI and RT+MEL groups compared with the RT group (*p* < 0.001) (Figure 2F).

### 2.3. Anti-Inflammatory and Anti-Fibrosis Effects of Amifostine and Melatonin After Radiation Exposure

**Figure 3:**
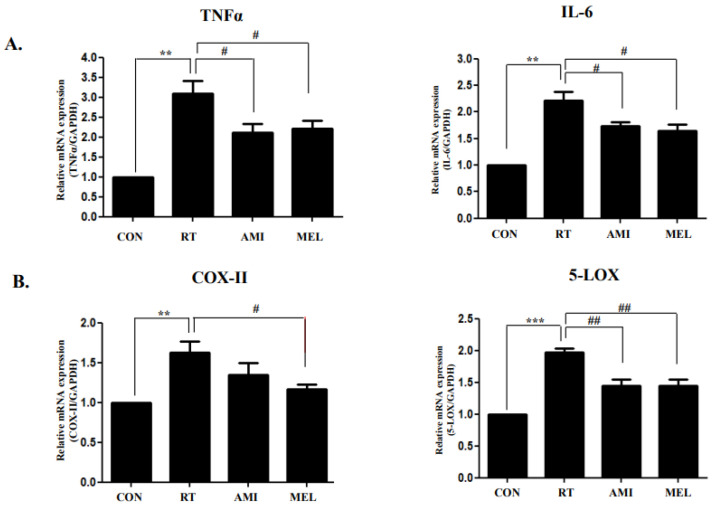
Inhibitory effect of AMI and MEL on inflammation. Submandibular gland inflammation increased in the RT group but decreased in the AMI and MEL groups. (**A**) mRNA expression of pro-inflammatory cytokines TNF-alpha and IL-6 was significantly upregulated in the RT group compared to the CON group (*p* < 0.01), whereas they were significantly downregulated in the RT+AMI and RT+MEL groups compared to the RT group (*p* < 0.05). (**B**) COX-II mRNA expression was significantly upregulated in the RT group compared with the CON group (*p* < 0.01), whereas it was downregulated in the RT+MEL group compared with the RT group (*p* < 0.05). 5-LOX mRNA expression was also significantly upregulated in the RT group compared with the CON group (*p* < 0.001), whereas it was significantly downregulated in the RT+AMI and RT+MEL groups compared with the RT group (*p* < 0.01). One-way ANOVA; ** *p* < 0.01, *** *p* < 0.001 vs. CON, # *p* < 0.05, ## *p* < 0.01 vs. RT.

In the RT group, mRNA expression of pro-inflammatory cytokines tumor necrosis factor alpha (TNFα) and interleukin 6 (IL-6) showed significant upregulation in the RT group compared to the CON group (*p* < 0.01), whereas they exhibited significant downregulation in the RT+AMI and RT+MEL groups compared to the RT group (*p* < 0.05) (Figure 3A). Similarly, cyclooxygenase-2 (COX-II) mRNA expression was significantly upregulated in the RT group compared with the CON group (*p* < 0.01) yet downregulated in the RT+MEL group compared with the RT group (*p* < 0.05). Moreover, 5-lipoxygenase (5-LOX) mRNA expression was significantly upregulated in the RT group compared with the CON group (*p* < 0.001), whereas it demonstrated significant downregulation in the RT+AMI and RT+MEL groups compared with the RT group (*p* < 0.01) (Figure 3B). These results underscore the anti-inflammatory effects of RT+AMI and RT+MEL treatment.

**Figure 4:**
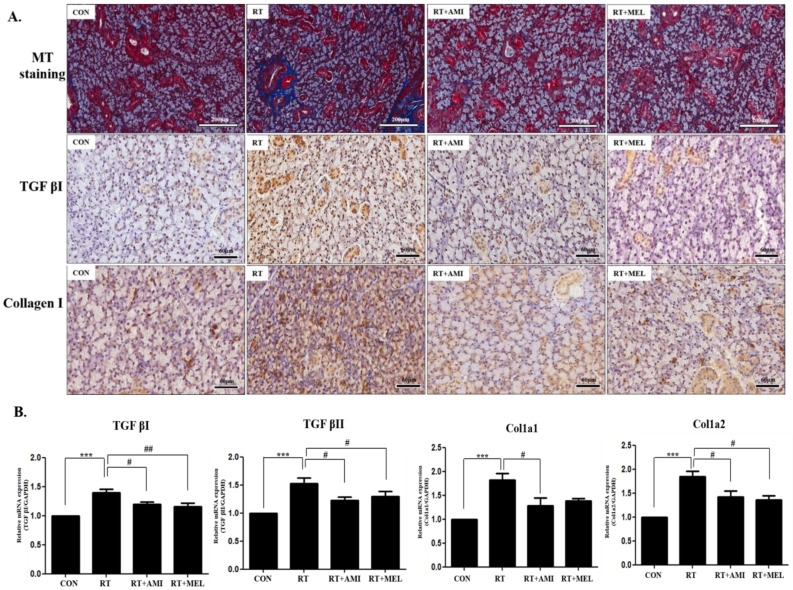
Suppressive effect of AMI and MEL on fibrosis. Submandibular gland fibrosis increased in the RT group but decreased in the AMI and MEL groups. (**A**) Masson’s trichrome staining to detect the fibrotic area showed peri-striated ductal and perivascular fibrosis increased in the RT group but decreased in the RT+AMI and RT+MEL groups (upper panel). TGF-β1-positive stained regions were significantly higher in the RT group, whereas they were lower in the RT+AMI and RT+MEL groups (middle panel). collagen-I-positive stained regions were significantly higher in the RT group but significantly reduced in the AMI and MEL groups (lower panel). (**B**) TGF-β1 and -β2 mRNA expression was significantly upregulated in the RT group compared with the CON group (*p* < 0.001), whereas they were downregulated in the RT+AMI (*p* < 0.05) and RT+MEL groups (TGF-βI; *p* < 0.01 and TGF-β2; *p* < 0.05) compared with the RT group. Col1a1 and Col1a2 mRNA expression was also significantly upregulated in the RT group compared with the CON group (*p* < 0.001), whereas they were downregulated in the RT+AMI (*p* < 0.05) and RT+MEL groups (Col1a2; *p* < 0.05) compared with the RT group. One-way ANOVA; *** *p* < 0.001 vs. CON, # *p* < 0.05, ## *p* < 0.01 vs. RT.

Additionally, in the RT group, there was a significant increase in the expression of transforming growth factor-beta 1 (TGF-β1) and collagen I, indicating elevated levels of fibrosis-related factors. Masson’s trichrome staining revealed an augmentation in peri-striated ductal and perivascular fibrosis in the RT group, which was markedly alleviated in the RT+AMI and RT+MEL groups (upper panel). TGF-β1-positive stained regions were significantly higher in the RT group, whereas they exhibited lower levels in the RT+AMI and RT+MEL groups (middle panel). Similarly, collagen-I-positive stained regions were significantly elevated in the RT group but significantly diminished in the RT+AMI and RT+MEL groups (lower panel) (Figure 4A). Moreover, TGF-β1 and -β2 mRNA expression levels were significantly upregulated in the RT group compared with the CON group (*p* < 0.001), whereas they were downregulated in the RT+AMI (*p* < 0.05) and RT+MEL groups (TGF-β1; *p* < 0.01 and TGF-β2; *p* < 0.05) compared with the RT group. Additionally, Col1a1 and Col1a2 mRNA expression levels were significantly upregulated in the RT group compared with the CON group (*p* < 0.001), whereas they were downregulated in the RT+AMI (*p* < 0.05) and RT+MEL groups (Col1a2; *p* < 0.05) compared with the RT group) (Figure 4B). These findings underscore the potent anti-fibrotic effects of AMI and MEL treatment.

### 2.4. Preventive Effect on Submandibular Gland Dysfunction

**Figure 5:**
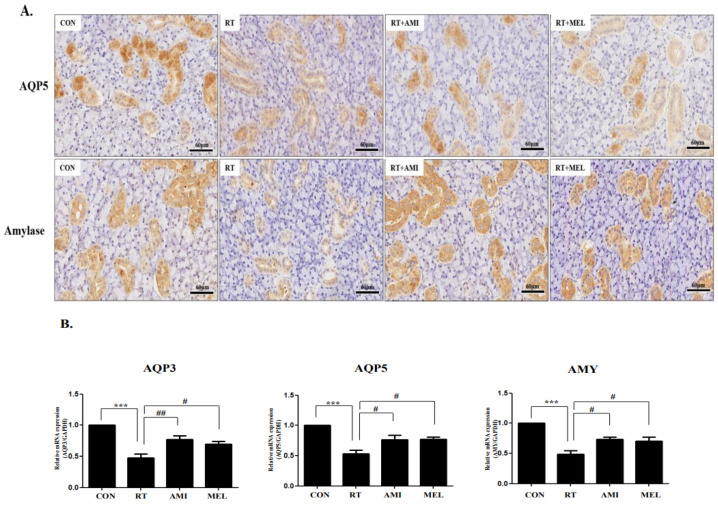
Improvement of submandibular gland function after treatment with AMI or MEL. Submandibular gland function decreased in the RT group but increased in the AMI and MEL treatment groups. (**A**) AQP5-positive stained regions were significantly lower in the RT group but increased in the RT+AMI and RT+MEL groups (upper panel). amylase-positive stained regions were also significantly lower in the RT group but higher in the RT+AMI and RT+MEL groups (lower panel). (**B**) AQP1 and AQP5 mRNA expression was significantly reduced in the RT group compared with the CON group (*p* < 0.001), whereas they were increased in the RT+AMI (AQP3; *p* < 0.001 and AQP5; *p* < 0.05) and RT+MEL groups (*p* < 0.05) compared with RT. AMY mRNA expression was also significantly reduced in the RT group compared with the CON group (*p* < 0.001), whereas it was elevated in the RT+AMI and RT+MEL groups (*p* < 0.05) compared to the RT group. One-way ANOVA; *** *p* < 0.001 vs. CON, # *p* < 0.05, ## *p* < 0.01 vs. RT.

Immunohistochemical staining revealed a decrease in aquaporin 5 (AQP5) and amylase expression after irradiation, which significantly increased following amifostine and melatonin treatment (Figure 5A). AQP5-positive stained regions were significantly lower in the RT group but increased in the RT+AMI and RT+MEL groups (upper panel). Similarly, amylase-positive stained regions were significantly lower in the RT group but higher in the RT+AMI and RT+MEL groups (lower panel). Moreover, mRNA expression levels of AQP3, AQP5, and amylase decreased post-radiation exposure but showed a significant increase in the treatment groups (Figure 5B). AQP1 and AQP5 mRNA expression was significantly reduced in the RT group compared with the CON group (*p* < 0.001), whereas they were increased in the RT+AMI (AQP3; *p* < 0.001 and AQP5; *p* < 0.05) and RT+MEL groups (*p* < 0.05) compared with RT. Furthermore, AMY mRNA expression was also significantly reduced in the RT group compared with the CON group (*p* < 0.001), whereas it was elevated in the RT+AMI and RT+MEL groups (*p* < 0.05) compared to the RT group. These findings underscore the effectiveness of amifostine and melatonin in preventing radiation-induced acute submandibular gland dysfunction.

### 2.5. Ferritinophagy

**Figure 6:**
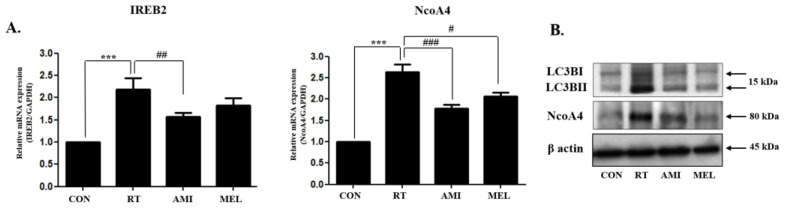
Effect of AMI and MEL on ferritinophagy. After irradiation, iron ferritinophagy was increased in the submandibular gland tissue and decreased in the AMI and MEL treatment groups. (**A**) IREB2 and NcoA4 mRNA expression was significantly upregulated in the RT group compared with the CON group (*p* < 0.001), whereas it was downregulated in the RT+AMI (IREB2; *p* < 0.01 and NcoA4; p,0.001) and RT+MEL groups (NcoA4; *p* < 0.05) compared with RT. (**B**) LCII (15kDa) and NcoA4 (80kDa) protein expression were significantly upregulated in the RT group and downregulated in the RT+AMI and RT+MEL groups. One-way ANOVA; *** *p* < 0.001 vs. CON, # *p* < 0.05, ## *p* < 0.01 and ### *p* < 0.001 vs. RT.

Ferritinophagy was explored to elucidate the mechanism underlying the increase in iron levels in the submandibular glands after irradiation. After irradiation, iron ferritinophagy was increased in the submandibular gland tissue but decreased in the AMI and MEL treatment groups. In the RT group, iron responsive element binding protein 2 (IREB2) and Nuclear Receptor Coactivator 4 (NcoA4) mRNA expression was significantly upregulated in the RT group compared with the CON group (*p* < 0.001), whereas it was downregulated in the AMI (IREB2; *p* < 0.01 and NcoA4; *p* < 0.001) and RT+MEL groups (NcoA4; *p* < 0.05) compared with RT. However, the expression of *Microtubule-associated proteins 1A/1B light chain 3B* (hereafter referred to as LC3) and NCOA4 was significantly decreased in the RT+AMI and RT+MEL groups compared with the RT group (Figure 6A,B). Furthermore, LC3B (15kDa) and NcoA4 (80kDa) protein expressions were significantly upregulated in the RT group and downregulated in the RT+AMI and RT+MEL groups (Figure 6A,B). These results underscore the potential of AMI and MEL in modulating ferritinophagy in the context of radiation-induced submandibular gland dysfunction. These findings suggest that irradiation-induced ferritinophagy contributes to the elevation of Fe^2+^ levels in the submandibular gland tissue.

## 3. Discussion

The initial cell and tissue damage caused by irradiation occurs because of the generation of large amounts of ROS by the conversion of water. ROS can destroy the cell structure by acting directly or indirectly on DNA, proteins, and lipids, resulting in apoptosis and necrosis [13]. Although excessive ROS can induce apoptosis and necrosis, they may also be associated with ferroptosis, a novel mechanism of cell death. The features of ferroptosis are ROS generation, the depletion of GPX4 in cells, lipid hydroperoxide accumulation, and the availability of iron [14]. Ferroptosis, caused by excessive ROS production after irradiation, has been proposed as a mechanism of irradiation-induced cell damage [3]. In this study, increased ROS, oxidized GSH, lipid peroxidation (4-HNE), and iron levels and decreased GPX4 were observed in salivary gland tissue after irradiation. Ferroptosis causes inflammation and fibrosis in the submandibular glands after radiation, ultimately resulting in salivary gland dysfunction (as shown by decreased AQP3, AQP5, and amylase expression in the salivary glands). These findings suggested that radiation-induced salivary gland dysfunction was caused by ferroptosis.

This study is the first to confirm that ferroptosis is involved in the salivary gland dysfunction that occurs after radiation. Amifostine is known to be an effective protective drug against radiation-induced xerostomia [6,7], however, although it is known to scavenge oxygen free radicals, the exact mechanism remains unclear. In this study, decreased iron levels, ROS production, oxidized GSH, and lipid peroxidation and increased GPX4 were observed in salivary gland tissues after amifostine treatment. In the RT+AMI group, inflammation and fibrosis were reduced and salivary gland function was improved. These findings suggest that the effect of amifostine on radiation-induced salivary gland dysfunction is related to the inhibition of radiation-induced ferroptosis by acting as a ROS scavenger. These results indicate that amifostine prevents xerostomia by inhibiting ferroptosis 10 days after radiation.

Although amifostine is effective against xerostomia after irradiation, its clinical application is limited owing to its severe side effects [8]. Regarding previous studies, there is still some controversy, as a recent randomized trial study found that amifostine has no preventive effect on xerostomia [15]. Moreover, the recent guideline stated that evidence remains insufficient for a recommendation for or against the use of amifostine during radiation therapy for head and neck cancer [16]. Therefore, a clinically safe and potent free radical scavenger may act as a novel salivary gland radioprotector. Melatonin acts as a circadian pacemaker and biological clock, however, it is a potent free radical scavenger [17,18]. A recent study reported that melatonin suppresses ferroptosis by regulating the SIRT6/p-Nrf2/GPX4 and SIRT6/NCOA4/FTH1 pathways in age-related cataract [19]. Similar to amifostine treatment, our results showed that melatonin treatment resulted in decreased iron levels, ROS production, lipid peroxidation, GSH, inflammation, and fibrosis and the recovery of salivary gland function. These findings imply that melatonin may play a role in preventing the decrease in salivary gland function after irradiation by inhibiting ferroptosis. These results are similar to those of previous studies showing that melatonin is beneficial in salivary gland functional recovery after irradiation [18]. Recent studies have shown that melatonin is effective in radiation-induced salivary gland dysfunction by inhibiting radiation-induced DNA damage and apoptosis and modulating oxidative stress [20]. However, no studies have shown that melatonin effectively inhibits ferroptosis in radiation-induced salivary gland dysfunction. Our study identified that melatonin preserves salivary gland function after irradiation through its anti-ferroptotic activity.

**Figure 7:**
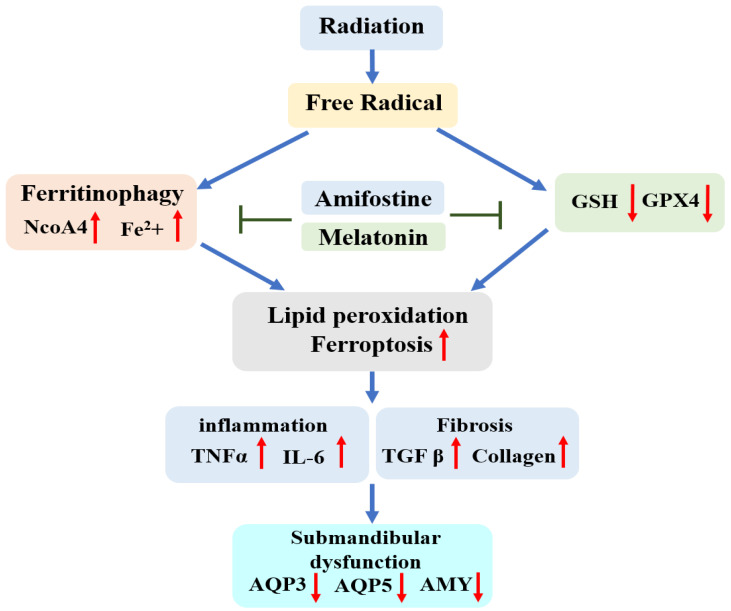
Summary of the study. Many free radicals generated by radiation increase iron concentrations in salivary glands via ferrotinophagy. Additionally, increased iron, excessive ROS, and decreased GPX4 levels cause lipid peroxidation (ferroptosis) in salivary gland tissue, ultimately leading to decreased salivary gland function. Salivary gland dysfunction occurs owing to radiation-induced ferrotinophagy and ferroptosis. Amifostine and melatonin inhibit radiation-induced ferroptosis and ferritinophagy and prevent salivary gland dysfunction after radiation.

Previous studies have shown that iron levels increase in the salivary glands after irradiation; however, the mechanism and significance of this increase are not yet clear [21,22]. Our study is the first to identify ferritinophagy as the mechanism by which iron levels increase in the salivary gland after radiation exposure. Ferritinophagy is a form of autophagy in which intracellular iron levels increase due to ferritin degradation and is thought to be associated with ferroptosis [23]. Increased Fe^2+^ levels are among the most important factors in ferroptosis. After irradiation, iron levels in the salivary gland tissue increased owing to ferritinophagy. The increase in iron levels induces ferroptosis, which leads to radiation-induced salivary gland dysfunction. Our results are similar to those of a study investigating NCOA4-mediated ferritinophagy in intestinal epithelial cells after radiation exposure [24]. As ROS scavengers, amifostine and melatonin not only inhibited ferroptosis but also inhibited ferroptosis by reducing NCOA4-mediated ferritinophagy (Figure 7).

There are many causes of salivary gland dysfunction, including irradiation, aging, menopause, drug treatment, and autoimmune diseases. Recently, ferroptosis was reported to be related to xerostomia that occurs after menopause [25] and that the salivary gland dysfunction caused by menopause improves when antiferroptotic agents are used [26,27]. In our study, ferroptosis was related to salivary gland dysfunction after radiation, and amifostine and melatonin, which have antioxidant scavenging effects, were found to have anti-ferroptotic activity. It is possible that amifostine and melatonin may also be effective in menopause-related xerostomia in which the ferroptosis mechanism is involved, however, this requires further investigation.

This study is the first to identify a relationship between radiation-induced acute salivary gland dysfunction and ferroptosis with ferritinophagy 10 days after radiation. It is also important to identify the mechanisms by which amifostine and melatonin inhibit ferroptosis and ferritinophagy induced 10 days after radiation to preserve acute salivary gland function. There are some limitations in directly applying these drugs to clinical practice using only in vivo studies on the acute response to radiation. Therefore, it is believed that future research on the exact mechanism and related molecules will be necessary through in vitro studies using 3D culture or organoids and in vivo studies on various tumor-cell-implanting syngeneic animal models [28,29,30].

However, this study also had some controversial findings. The effects of irradiation on cancer cell death are associated with ferroptosis, therefore, it is possible that amifostine and melatonin not only reduce xerostomia caused by radiation but also reduce the therapeutic effect of radiation [31]. It is essential to investigate the treatment time, injection method, and concentration of amifostine and melatonin that can prevent a reduction in salivary gland function owing to irradiation without interfering with the therapeutic effect.

This study investigated the acute response of salivary glands 10 days after radiation and the effects and mechanisms of amifostine and melatonin. However, there is not enough information to make overall conclusions regarding the effects of early radiation. Therefore, future studies are needed to evaluate the effects of amifostine and melatonin on radiation-induced acute and chronic salivary gland dysfunction at pre-treatment time points and a post-treatment time point of 10 days.

There is also a need to identify the mechanism for later changes in the salivary glands due to irradiation and to study the long-term effects and safety of these drugs. Additionally, this study did not directly evaluate the amount of saliva in the evaluation of salivary gland dysfunction. Salivary gland function was indirectly confirmed through the evaluation of aquaporin and amylase expression. Future research is needed on chronic response and direct salivary gland function due to irradiation. Nevertheless, in the absence of a standard treatment for dry mouth by radiation therapy, this study is expected to help understand the mechanism of salivary gland dysfunction by radiation therapy and develop a treatment for dry mouth by radiation therapy.

## 4. Materials and Methods

### 4.1. Animals

Sprague Dawley rats were procured from Central Lab (Seoul, Republic of Korea) and housed in a pathogen-free facility under a 12-hour light/dark cycle, with access to standard rat chow and water ad libitum. The rats were randomly divided into four groups: control (CON, n = 6), irradiation (RT, n = 6), irradiation with amifostine (RT+AMI, n = 6), and irradiation with melatonin (RT+MEL, n = 6). Weight matching (200 ± 25 g) was performed for each group after a one-week acclimation period at the initiation of the study.

Rats in the irradiation group were exposed to a single dose of 15 Gy directed to the head and neck region. With a rat mounted in the prone position, a single dose of 15 Gy was delivered to the head at 5 Gy/min using an AP-PA 6 MV photon beam generated by a linear accelerator (Versa HD, Elekta, Stockholm, Sweden). A square radiation field (5 cm) shaped with a multi-leaf collimator was applied. To ensure uniform dose distribution, a tissue-equivalent bolus was placed on the head. Amifostine (200 mg/kg, administered intraperitoneally) and melatonin (100 mg/kg, administered intraperitoneally) were given to the respective treatment groups 30 min prior to irradiation.

Sacrifice of the rats occurred 10 days post-irradiation. The experimental protocol was approved by the Institutional Animal Care and Ethics Committee of Pusan National University Hospital (Approval No. PNUH-2022-200).

### 4.2. Tissue Extract Preparation

The frozen submandibular gland tissue underwent homogenization in a hypotonic lysis buffer (buffer A) containing 10 mM KCl, 2 mM MgCl_2_, 1 mM dithiothreitol, 0.1 mM ethylenediaminetetraacetic acid (EDTA), 0.1 mM phenylmethylsulfonyl fluoride, 1 mM pepstatin, 2 mM leupeptin, 20 mM β-glycerophosphate, 20 mM NaF, 2 mM Na3VO4, and 10 mM HEPES at pH 7.4. This homogenization was performed using a tissue homogenizer (Homogenizing Stirrer, Daihan Science, HS-30E, Seoul, Republic of Korea) for 20 s. The volume of the buffer was determined by measuring the tissue weight in triplicate. After cooling the homogenates on ice for 15 min, 125 mL of 10% Nonidet P-40 (NP-40) solution was added, mixed for 15 s, and then centrifuged at 14,000× *g* for 2 min. The resulting supernatants were stored at −80 °C and subsequently utilized as the total cellular extraction. Protein concentration was assessed through a bicinchoninic acid assay (BCA, Life Technologies, Carlsbad, CA, USA).

### 4.3. Staining and Immunohistochemical Analysis

The submandibular gland from each rat was isolated and fixed in 4% formalin overnight. Paraffin embedding was carried out using an automatic tissue processor (Leica TP1020; Leica, Wetzlar, Germany) and a heated paraffin embedding module (Leica EG1150H, Leica, Wetzlar, Germany). Deparaffinized sections of 8 μm thickness were mounted on glass slides and subjected to hematoxylin and eosin staining as well as Masson’s trichrome staining. A Prussian blue stain reaction in which ionic iron reacts with acid ferrocyanide produces a blue color was carried out. Any ferric ion present in the tissue combines with the ferrocyanide and results in the formation of a bright blue pigment called Prussian blue, or ferric ferrocyanide. Deparaffinized and rehydrated sections were incubated for 3 min in iron stain (mix of potassium ferrocyanide and hydrochloric acid) and rinsed in water. Then, samples were counterstained in nuclear fast red and dehydrated in alcohol and mounted. Immunohistochemistry was performed on deparaffinized sections incubated with primary antibodies targeting 4-hydroxynonenal (1:1000; Abcam, Cambridge, UK), transforming growth factor β1 (TGF-β1; 1:2000; Santa Cruz Biotechnology, Dallas, TX, USA), collagen I (1:1000; Abcam), aquaporin 5 (AQP5; 1:1000; Abcam), and amylase (1:1000; Cell Signaling Technology, Danvers, MA, USA) at 4 °C for 24 h. Following primary antibody incubation and rinsing, sections were treated with goat anti-rabbit secondary antibodies (1:1000; ENZO Biochem, New York, NY, USA) for 1 h at room temperature and double-stained with 3,3′-diaminobenzidine. Negative controls were prepared by omitting the primary antibody and incubating sections in phosphate-buffered saline supplemented with 1% bovine serum albumin. Representative images were captured at 20x magnification using a light microscope (ScanScope^®^ CS System, CA, USA). Morphometric analysis of whole submandibular gland tissue sections was conducted using 20x magnified images and image analysis system software (Aperio ImageScope v12.4.6; Leica Wetzlar, Germany).

### 4.4. Quantitation of Redox Status and Ferroptosis Response

ROS generation was assessed using a fluorescent probe. Specifically, 2′,7′-dichlorofluorescin diacetate was added to achieve a final concentration of 25 mM in a total volume of 250 mL homogenate. The fluorescence intensity was recorded every 5 min over a 30-minute period using a fluorescence plate reader, with excitation and emission wavelengths set at 485 nm and 530 nm, respectively. Total cellular iron (Fe^2^⁺ and Fe^3^⁺) concentrations in submandibular gland tissues were determined using a rat-specific colorimetric iron assay kit (Biovision Inc., Spring Valley, CA, USA). In brief, ferric carrier proteins facilitated the release of ferric ions into a solution in the presence of an acid buffer. Following reduction to the ferrous state (Fe^2+^), Fe reacted with Ferene S to form a stable complex, detectable by absorbance at 593 nm. The activity of glutathione peroxidase 4 (GPX4) was evaluated using a GPX4 assay kit (Abcam, Cambridge, MA, USA), as per the manufacturer’s instructions. To measure glutathione (GSH) levels, 1 mM EDTA–50 mM phosphate buffer was added to the supernatant of trichloroacetic acid (TCA)-treated homogenates, followed by o-phthaldehyde, and incubated for 25 min at room temperature. For assessing oxidized GSH levels, N-ethylmaleimide was added to the supernatant of TCA-treated homogenates. After 30 min at room temperature, 0.5 N NaOH and o-phthaldehyde were added to the samples and incubated for 25 min. GSH and oxidized GSH levels were quantified at excitation and emission wavelengths of 360 nm and 460 nm.

### 4.5. Quantitative Real-Time PCR

Total RNA was extracted from tissue using TRIzol reagent (Thermo Fisher Scientific, Waltham, MA, USA) following the manufacturer’s protocol. The concentration of RNA was quantified using a NanoDrop^®^ ND-1000 spectrophotometer (Thermo Fisher Scientific), and the 260 nm/280 nm absorbance ratio was utilized to assess RNA purity. Reverse transcription was conducted using a reverse transcription kit (Thermo Fisher Scientific) according to the manufacturer’s instructions. RNA concentration and purity were reassessed using the NanoDrop^®^ ND-1000 spectrophotometer with the 260 nm/280 nm absorbance ratio. Quantitative polymerase chain reaction (qPCR) was carried out following the SYBR^®^ Green PCR protocol (Thermo Fisher Scientific). The reaction conditions included an initial denaturation step at 95 °C for 10 min (one cycle), followed by 40 cycles of denaturation at 95 °C for 10 s and annealing/extension at 60 °C for 30 s. The ABI PRISM 7900 HT Sequence Detection System (Thermo Fisher Scientific) was employed to continuously measure gene-specific PCR products. Primer sequences used are detailed in Table 1. The delta CT/target gene delta CT ratio was calculated by normalizing the cycle threshold differences (delta CT) with GAPDH expression levels.

### 4.6. Western Blotting

Protein assay was determined using a BCA protein assay kit (Thermo Fisher Scientific). Equal amounts of total protein were loaded onto 10% acrylamide gels for sodium dodecyl sulfate-polyacrylamide gel electrophoresis and subsequently transferred to polyvinylidene fluoride membranes. The membranes were then blocked in a buffer containing 5% bovine serum albumin for 1 h. Incubation with primary antibodies against LC3B (15kDa, 1:1000; Abcam) and nuclear receptor coactivator 4 (80 kDa, NCOA4; 1:2000; Cell Signaling Technology) was carried out overnight at 4 °C. An anti-β-actin antibody (45 kDa, 1:2000; Santa Cruz Biotechnology) served as an internal control. Following three 10-minute washes, the membranes were incubated with peroxidase-conjugated secondary antibodies (1:10,000 dilution for both anti-mouse and anti-rabbit antibodies) for 1 h at 21 °C. Visualization of the results was achieved using West-Zol Plus and chemiluminescent FluorchemTM SP (Alpha Innotech Corporation, San Leandro, CA, USA).

### 4.7. Statistical Analysis

All quantitative data are expressed as the mean ± standard error of the mean (SEM) from a minimum of three independent experiments. Statistical significance between groups was determined using one-way analysis of variance (ANOVA), with *p* < 0.05 considered as statistically significant.

## 5. Conclusions

Radiation-induced salivary gland dysfunction is associated with ferroptosis and ferritinophagy. Amifostine and melatonin inhibited radiation-induced ferroptosis and ferritinophagy and prevented acute salivary gland dysfunction 10 days after radiation.

## Figures and Tables

**Table 1:** Primer sequences of the different target genes analyzed using PCR.

Gene	Direction	Sequence
TNFɑ	Forward	GGTCAACCTGCCCAAGTACT
NM_012675	Reverse	CTCCAAAGTAGACCTGCCCG
IL-6	Forward	ATCTGCCCTTCAGGAACAGC
NM_012589	Reverse	GAAGTAGGGAAGGCAGTGGC
COX-II	Forward	GGTTCACCCGAGGACTGGGC
NM_017232	Reverse	CGCAGGTGCTCAGGGACGTG
5-LOX	Forward	ATTGTTCCCATTGCCATCCAGCTCA
NM_009662	Reverse	TCGTTCTCATAGTAGATGCTCACCA
TGF-β1	Forward	GACGTTCGCCATAACCAAGT
NM_021578	Reverse	CTGCAGGTTCTCAATGCAAA
TGF-β2	Forward	CCAATCACGCAATAGTTCTGG
NM_031131	Reverse	CGCTGTATCGTATGGCGAT
Col1a1	Forward	CAGGATGCAGTCCCTGAAAT
NM_053304	Reverse	GAGGTGGCCTAGGTGGTGTA
Col1a2	Forward	GGTCAGCACCACCGATGTC
NM_053356	Reverse	CACGCCTGCCCTTCCTT
AQP-3	Forward	AATTGTCTGGAGCCCACTTG
NM_031703	Reverse	CAGCTTGATCCAGGGCTCTC
AQP-5	Forward	CATGAACCCAGCCCGATCTT
NM_012779	Reverse	AGAAGACCCAGTGAGAGGGG
AMY	Forward	GCAACCAAGTAGCTTTTGGCA
NM_001010970	Reverse	TGCCATCGACTTTGTCTCCAG
IREB2	Forward	GGGAATTCTTGGGTGGGGAG
NM_022863	Reverse	AACAAACTTTCCAGCCACGC
NcoA4	Forward	AGACTACGGCTCCTGCTA
NM_001034007	Reverse	CTGACTAAGGTTTCCCACT
GAPDH	Forward	ACCCCCAATGTATCCGTTGT
NM_017008	Reverse	TACTCCTTGGAGGCCATGTA

## Data Availability

All relevant data are contained within the manuscript.
